# Supplementary material for: Scalable genotyping in fixed transcriptomes resolves clonal heterogeneity via single-cell sequencing
Source: bioRxiv. 2026 Jul 3:2026.04.11.717967. Originally published 2026 Apr 12. Preprint. [Version 3] doi: 10.64898/2026.04.11.717967 (PMC13081950; doi:10.64898/2026.04.11.717967)
Supplement: 1 [file NIHPP2026.04.11.717967V3-supplement-1.pdf]

bioRxiv preprint doi: https://doi.org/10.64898/2026.04.11.717967; this version posted July 3, 2026. The copyright holder for this preprint (which was not certified by peer review) is the author/funder, who has granted bioRxiv a license to display the preprint in perpetuity. It is made available under a CC-BY 4.0 International license.

SUPPLEMENTARY FIGURES

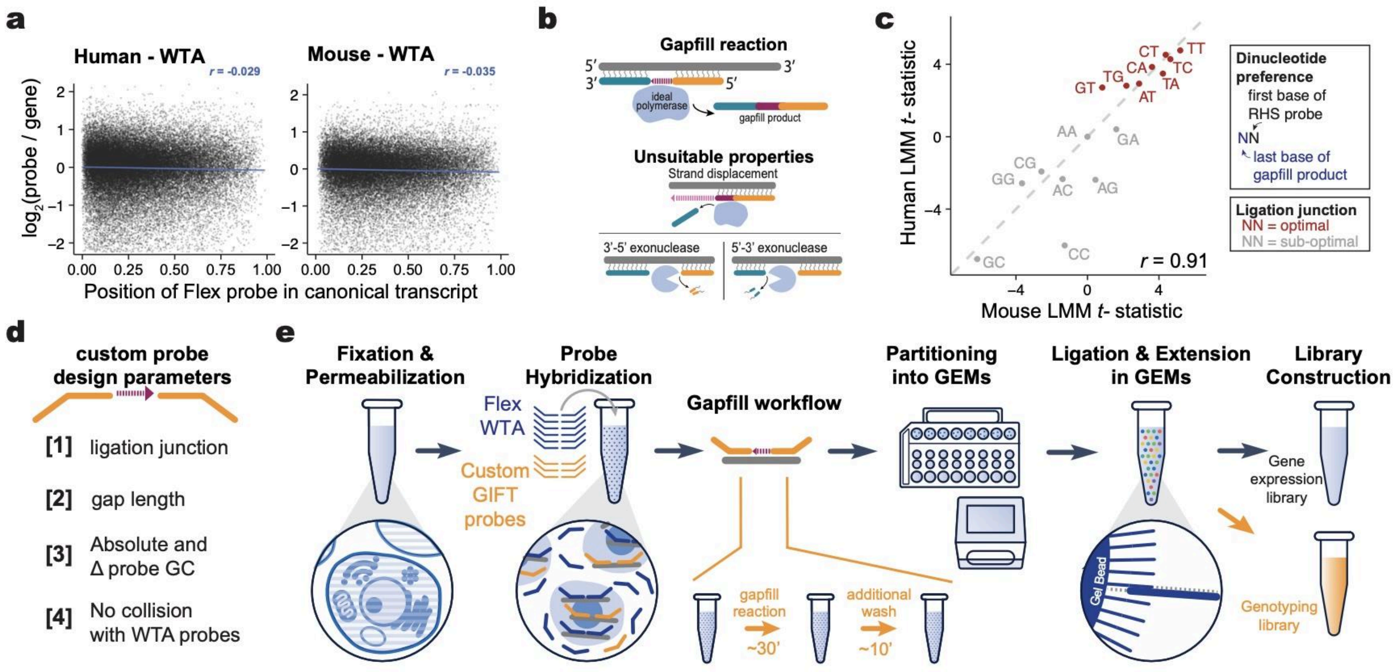

Supplementary Figure 1. Development of GIFT. a, Normalized probe count vs. transcript position for Flex WTA panels showing that probe-based detection is possible throughout the transcript (n=53,330 human; 54,263 mouse probe pairs). **b**, A suitable gapfill polymerase is strand displacement deficient and lacks exonuclease activity. **c**, Summary of linear mixed model (LMM) associations for human and mouse probe sets to define optimal ligation junctions based on the Flex WTA probesets for mouse and human. **d**, Parameters used for custom GIFT probe design. **e**, Detailed overview of flex plus gapfill (GIFT) workflow. Separate PCR handles are used to separately amplify gene expression (WTA) and genotyping (GIFT) libraries.

bioRxiv preprint doi: https://doi.org/10.64898/2026.04.11.717967; this version posted July 3, 2026. The copyright holder for this preprint (which was not certified by peer review) is the author/funder, who has granted bioRxiv a license to display the preprint in perpetuity. It is made available under a CC-BY 4.0 International license.

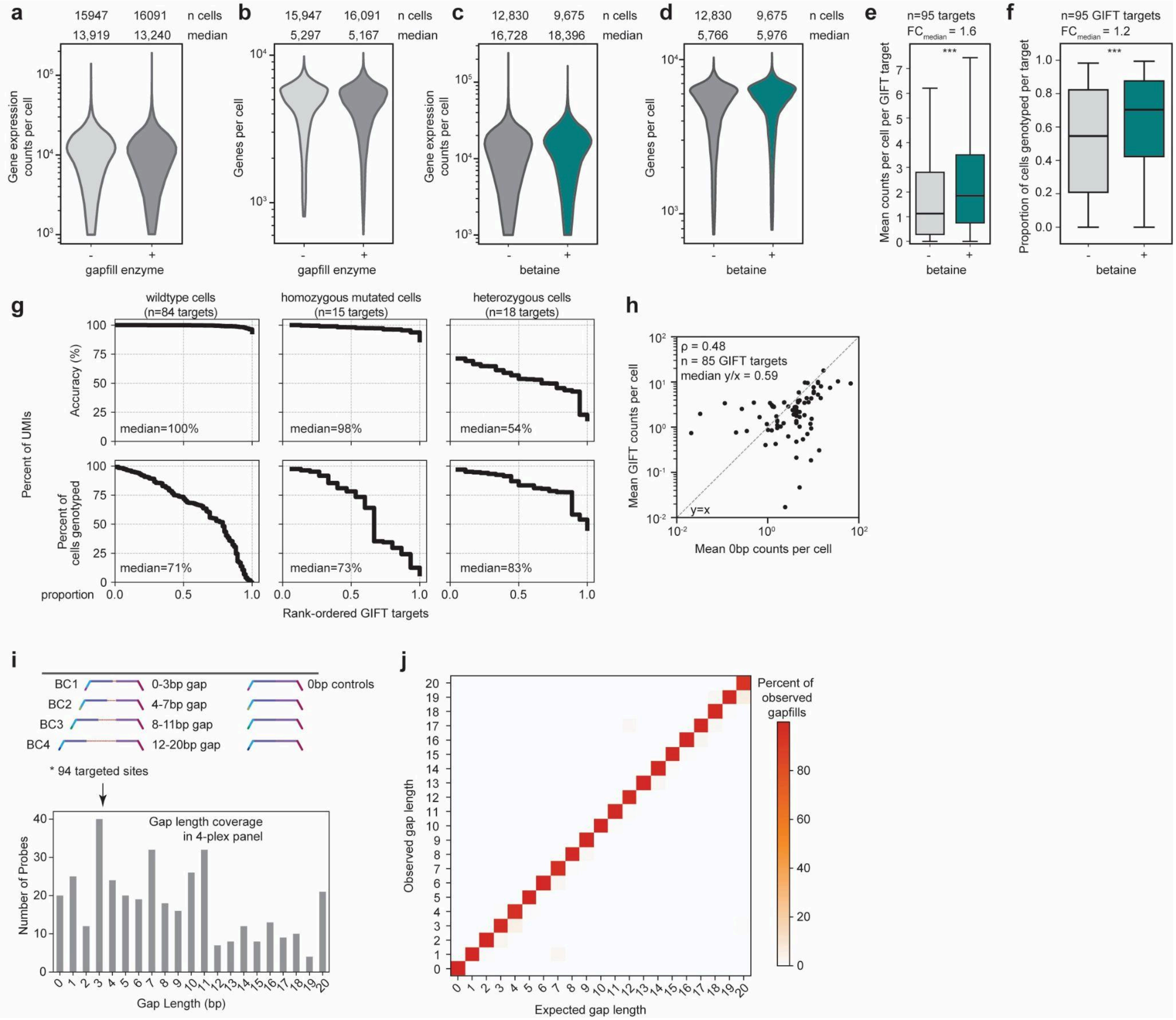

Supplementary Figure 2. GIFT metrics in cell lines. a-b, The number of counts (**a**) or genes (**b**) captured per cell for Flex gene expression assay (WTA) is similar with or without the gapfill polymerase. The median number of counts per cell with gapfill enzyme is 95% of the median per cell without (one-sided Mann-Whitney U; $p < 0.0005$). The median number of genes captured per cell with gapfill enzyme is 97.5% of the median per cell without (one-sided Mann-Whitney U; $p < 0.0005$). Other GIFT steps were included for both conditions with only polymerase omitted for "- enzyme". Sequencing saturation was 15% (with enzyme) or 14% (without enzyme). **c-d**, The number of counts (**c**) or genes (**d**) captured per cell for gene expression (WTA) is similar with or without betaine. The median number of counts per cell with betaine is 110% of the median per cell without ($p < 0.0005$, one-sided Mann-Whitney U test). The median number of genes captured per cell with betaine is 104% of the median per cell without ($p < 0.0005$, one-sided Mann-Whitney U test). Sequencing saturation was 18% (with betaine) or 16% (without betaine). **e-f**, Genotyping yields for GIFT carried out with or without betaine, reported as mean counts per cell per target (**e**) and proportion of cells genotyped per target (**f**, ≥1 count per cell). Betaine increases yield ($p < 0.0005$, one-sided Wilcoxon test). The same GIFT targets were used for both experiments. **g**, Cell genotyping metrics across all targets in the cell line mixing experiment shown in **Fig. 1**. *Top*: Genotyping accuracy for genotyped cells (≥1 GIFT

bioRxiv preprint doi: https://doi.org/10.64898/2026.04.11.717967; this version posted July 3, 2026. The copyright holder for this preprint (which was not certified by peer review) is the author/funder, who has granted bioRxiv a license to display the preprint in perpetuity. It is made available under a CC-BY 4.0 International license.

count). *Bottom*: Proportion of cells for which GIFT assigns a genotype. **h**, GIFT counts per cell versus gene-matched 0-bp control probes (ρ, Spearman correlation; y/x measures gapfill efficiency). Control probes are targeted elsewhere on the same transcript but have no gap between probes. **i**, Design of gap length experiment. *Top*: Probe design strategy for a representative targeted site. The gap lengths for each probe barcode varied by target; for example, some targets had the longest gap on BC1 or BC2 or BC3 rather than BC4 as depicted for the target shown. *Bottom*: Number of probes for each gap length. Full panel is in **Extended Data Table 1b**. **j**, Observed vs. expected gap lengths for experiment described in **i**.

bioRxiv preprint doi: https://doi.org/10.64898/2026.04.11.717967; this version posted July 3, 2026. The copyright holder for this preprint (which was not certified by peer review) is the author/funder, who has granted bioRxiv a license to display the preprint in perpetuity. It is made available under a CC-BY 4.0 International license.

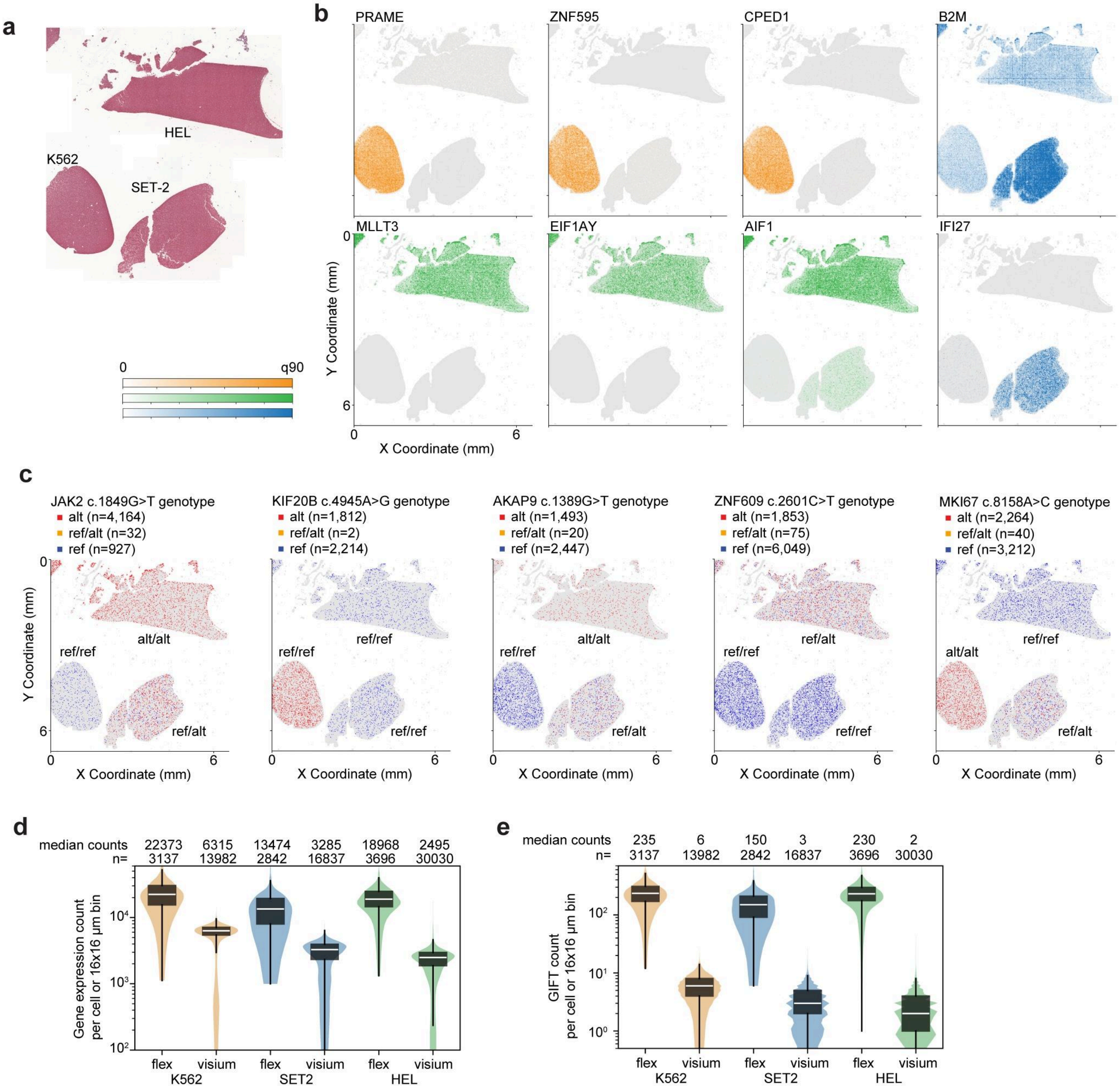

Supplementary Figure 3: GIFT in Visium HD. a, H&E staining for spatial GIFT showing locations of cells in the capture area. **b**, Cell type-specific marker gene expression confirming cell identity for the patterning of the cell lines. q90, 90th percentile of normalized counts per bin. **c,** Spatial genotyping showing the captured alleles in each 16 x 16 µm bin. Expected genotypes by cell line are annotated next to the spatial region. **d-e**, Comparison of gene expression (**d**) and GIFT genotyping (**e**) yields per cell or 16 x 16 µm bin from Flex or Visium HD, respectively, stratified by cell line. The same GIFT targets were included for both experiments (n=84 shared targets).

bioRxiv preprint doi: https://doi.org/10.64898/2026.04.11.717967; this version posted July 3, 2026. The copyright holder for this preprint (which was not certified by peer review) is the author/funder, who has granted bioRxiv a license to display the preprint in perpetuity. It is made available under a CC-BY 4.0 International license.

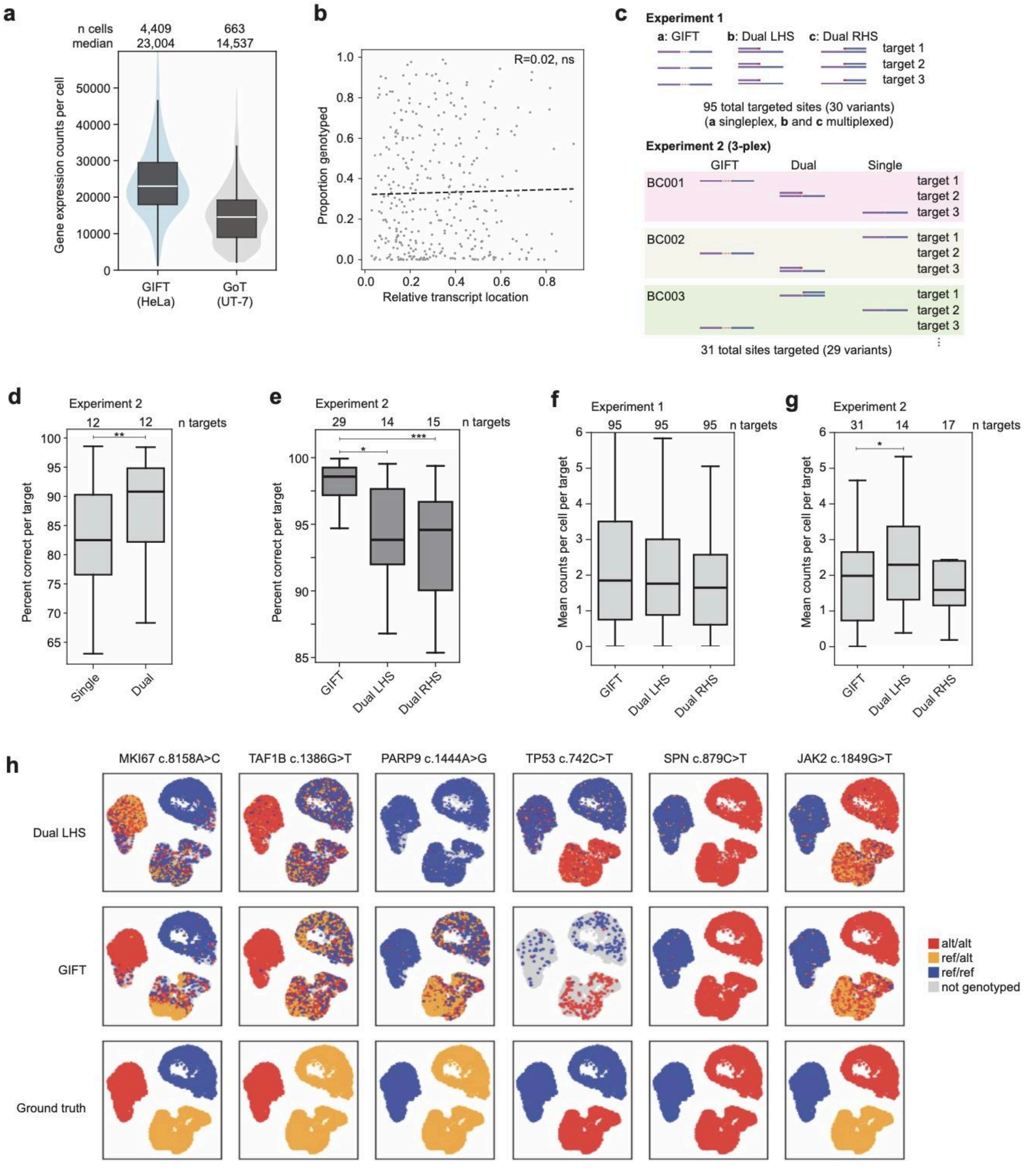

Supplementary Figure 4: GIFT benchmarking in cell lines. a-b, Comparisons between GIFT (this work) and GoT[10]. **a**, Gene expression counts per cell for GIFT vs GoT for 17,225 genes captured in both experiments. **b**, GIFT genotyping performance is not dependent on transcript location (R, Pearson correlation; p-value=0.7, n=344 GIFT targets). **c-h**, Benchmarking of GIFT (gapfill) against dual probe and single probe genotyping. **c**, Overview of two experiments. Genotyping accuracy for experiment 1 is shown in **Fig. 2f,g**. **d-e**, Percent of genotyping counts (UMIs) that are correct for each target by method (Dual vs. Single probe genotyping in **d** and GIFT vs. Dual in **e**). Targets are shared between GIFT and Dual probes but

bioRxiv preprint doi: https://doi.org/10.64898/2026.04.11.717967; this version posted July 3, 2026. The copyright holder for this preprint (which was not certified by peer review) is the author/funder, who has granted bioRxiv a license to display the preprint in perpetuity. It is made available under a CC-BY 4.0 International license.

are mutually exclusive for Dual LHS and Dual RHS (*** $p < 0.0005$, ** $p < 0.005$, * $p < 0.05$, one-sided Wilcoxon test). **f-g**, Mean counts per cell per target by genotyping strategy for each experiment (* $p < 0.05$, one-sided Wilcoxon test). **h**, UMAP embeddings of representative variants, colored by Dual LHS, GIFT, or ground truth (bulk) genotypes (n = 9,675 GIFT-assayed cells, n = 16,097 Dual probe-assayed cells).

bioRxiv preprint doi: https://doi.org/10.64898/2026.04.11.717967; this version posted July 3, 2026. The copyright holder for this preprint (which was not certified by peer review) is the author/funder, who has granted bioRxiv a license to display the preprint in perpetuity. It is made available under a CC-BY 4.0 International license.

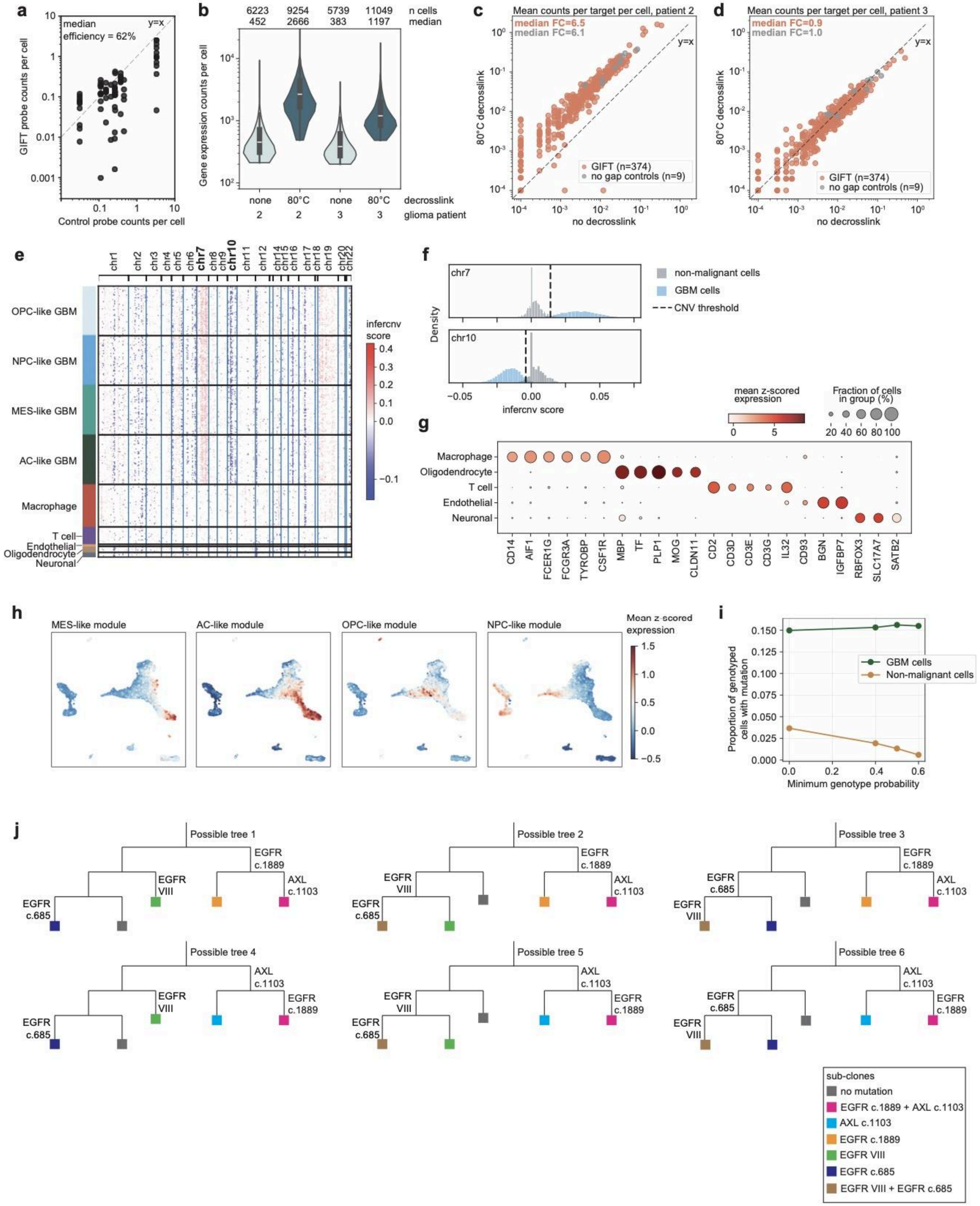

Supplementary Figure 5: GIFT genotyping in archival FFPE tissue. a, GIFT counts per cell versus gene-matched 0-bp control probes for FFPE GBM tissue (80° dcl, n=72 GIFT targets with matched controls),

bioRxiv preprint doi: https://doi.org/10.64898/2026.04.11.717967; this version posted July 3, 2026. The copyright holder for this preprint (which was not certified by peer review) is the author/funder, who has granted bioRxiv a license to display the preprint in perpetuity. It is made available under a CC-BY 4.0 International license.

as shown for cell lines in **Supplementary Fig. 2h**. This is for the same patient as in **Fig. 3**, hereafter called patient 1. Gapfill efficiency is the ratio of GIFT to control counts (y/x). Control probes are targeted elsewhere on the same transcript but have no gap between probes. **b-d**, Effect of decrosslinking on gene expression (WTA, **b**) and GIFT genotyping (**c-d**) yields in 2 additional glioma patients. Decrosslinking improves gene expression yield per cell for all patients. Decrosslinking improves GIFT yield per cell for patient 2. For patient 3, decrosslinking does not improve GIFT yield per cell but also does not improve yields for the 0-bp control probes, despite improvements for WTA. **e**, Inferred CNVs for GBM patient 1. GBM cells show gain of chromosome 7 and loss of chromosome 10. NPC, neural progenitor cell; AC, astrocyte; MES, mesenchymal; OPC, oligodendrocyte progenitor cell. **f**, Distributions of infercnv scores for chromosome 7 (*top*) and chromosome 10 (*bottom*) for GBM and non-malignant cells. Thresholds were added by visual inspection and used to classify cells in **Fig. 3g**. **g**, Z-scored, log-normalized expression of marker genes in non-malignant cell types. **h**, Expression of GBM subtype modules[18]. NPC, neural progenitor cell; AC, astrocyte; MES, mesenchymal; OPC, oligodendrocyte progenitor cell. **i**, Proportion of genotyped cells with at least one mutation based on genotype calls at different probability thresholds. As the minimum genotype probability increases, the false positive rate of mutation in non-malignant cells decreases. The genotype probability is defined as the probability that a cell is homozygous mutated, heterozygous, or wildtype, but homozygous and heterozygous mutation calls are included when calculating the proportion mutated. **j**, Expansion of possible phylogenies encompassed by the minimal tree shown in **Fig. 3h**. Our assumptions for defining possible lineage trees are detailed in methods.

bioRxiv preprint doi: https://doi.org/10.64898/2026.04.11.717967; this version posted July 3, 2026. The copyright holder for this preprint (which was not certified by peer review) is the author/funder, who has granted bioRxiv a license to display the preprint in perpetuity. It is made available under a CC-BY 4.0 International license.

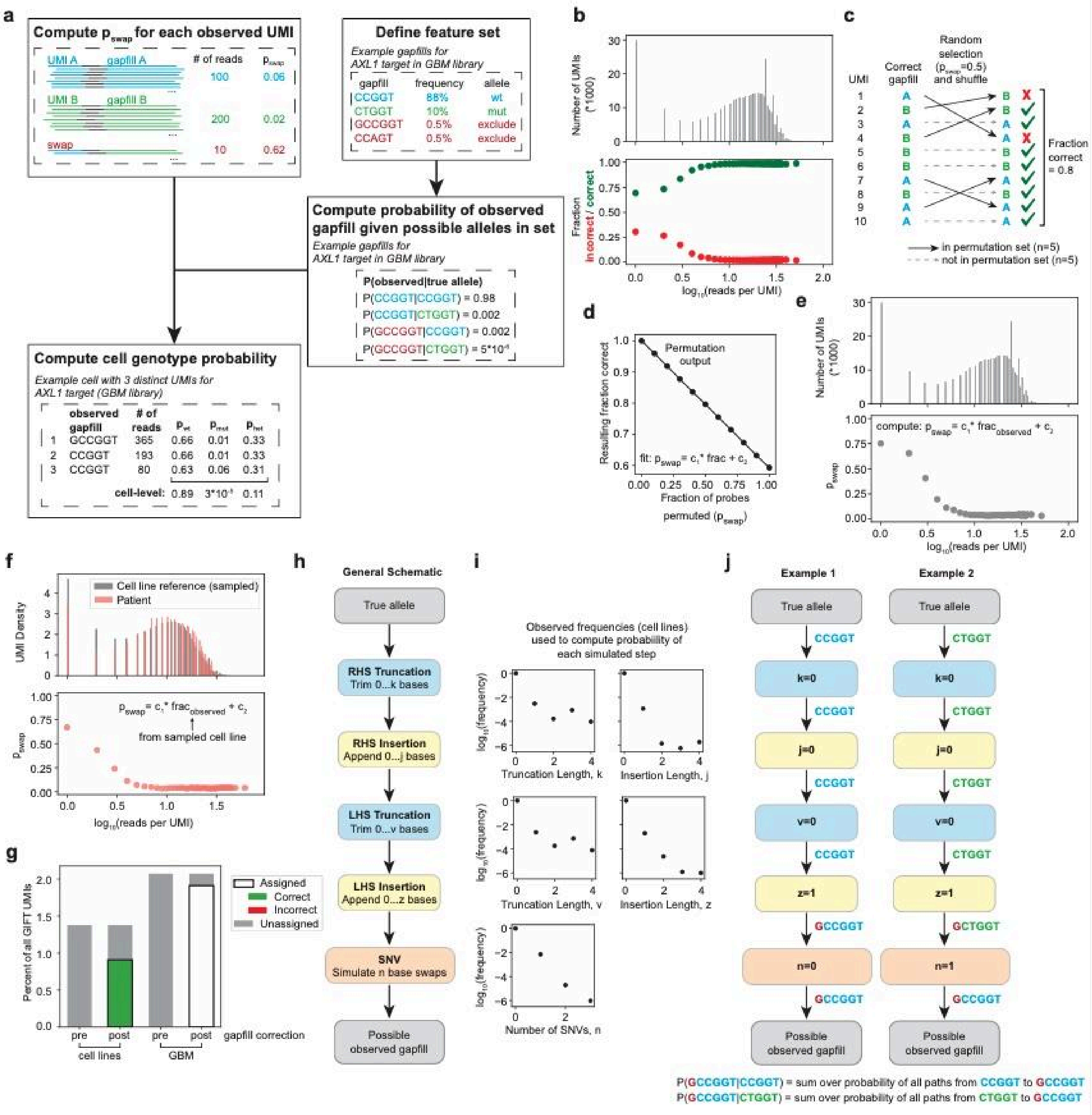

Supplementary Figure 6: Probabilistic model for assigning genotypes from GIFT. a, Our probabilistic strategy for assigning genotypes from GIFT counts (**methods**). In the first step, we use the number of reads per UMI to compute the probability (p_{swap}) that a UMI is an artifact of PCR-mediated template switching. An example of a swapped product with few reads is shown. In the second step, we define a constrained feature set that excludes observed gapfills without significant signal above noise. In the third step, we compute for each observed gapfill the probability of observation given possible true alleles in the set. For gapfills that are not included in the set, such as the example shown in red, it is still much more likely to come from one possible allele than the other. In the fourth step, we integrate information across UMIs for the same target in each cell to compute probabilities that a cell is wildtype, homozygous mutated, or heterozygous. An example

bioRxiv preprint doi: https://doi.org/10.64898/2026.04.11.717967; this version posted July 3, 2026. The copyright holder for this preprint (which was not certified by peer review) is the author/funder, who has granted bioRxiv a license to display the preprint in perpetuity. It is made available under a CC-BY 4.0 International license.

calculation for a cell with 3 observed *AXL1* counts is shown. **b-f**, Details of p_{swap} calculation. **b**, *Top*: Distribution of reads per UMI for a representative cell line sample. *Bottom*: Probability that a UMI is correct (matches ground truth genotype) or incorrect as a function of reads per UMI. **c**, Permutation approach used to infer p_{swap} from the observed fraction correct. We took all UMIs in the dataset and shuffled the genotypes of a random selection of UMIs. The proportion of UMIs selected for shuffling is a proxy for p_{swap}. This example shows a set of 10 UMIs permuted at p_{swap} = 0.5. **d**, Linear regression relating p_{swap} to the resulting fraction correct from the synthetic permutations. This equation can then be used to compute p_{swap} from the observed fraction correct in a real dataset. **e**, *Top*: Distribution of reads per UMI in the cell line dataset as in **b**. *Bottom*: Inferred p_{swap} computed from the fraction correct values in **b** and the regression fit in **d**. **f**, Inference of p_{swap} for patient data using the values computed for the cell line data. The cell line reference is sampled to closely match the distribution of reads per UMI for the patient dataset, which accounts for differences in sequencing depth. Then, the cell line fraction correct at each sampled read count is used to compute p_{swap} for the patient UMIs. **g-j**, Details of edit distance calculations used to compute the probability of an observed gapfill given possible true alleles. **g**, Breakdown of GIFT UMIs requiring gapfill correction, which is detailed in subsequent panels. Before gapfill correction, unassigned UMIs are any gapfills that do not exactly match an allele in the feature set. After gapfill correction, assigned UMIs are those for which we can assign an allele with >90% confidence. In cell lines, we confirm that accuracy in this set is high (99.8% correct). **h-i**, Simulated steps to model the possible alterations in a gapfill sequence that could occur from the true allele template to the observed sequence (**h**). These changes could occur during the gapfill polymerase step, PCR, or sequencing, and we determine their rates empirically based on observed gapfill sequences in cell line datasets (**i**). We compute the probability of each observed sequence using the product of the inferred probabilities at each step. **j**, Examples of edit distance calculation for an observed gapfill (GCCGGT) and two possible true alleles in the feature set. Example 1 (CCGGT) requires fewer changes to the original sequence, which is why this is much more likely to be the true allele, as shown by the calculation of P(observed|true allele) in **a**.

bioRxiv preprint doi: https://doi.org/10.64898/2026.04.11.717967; this version posted July 3, 2026. The copyright holder for this preprint (which was not certified by peer review) is the author/funder, who has granted bioRxiv a license to display the preprint in perpetuity. It is made available under a CC-BY 4.0 International license.

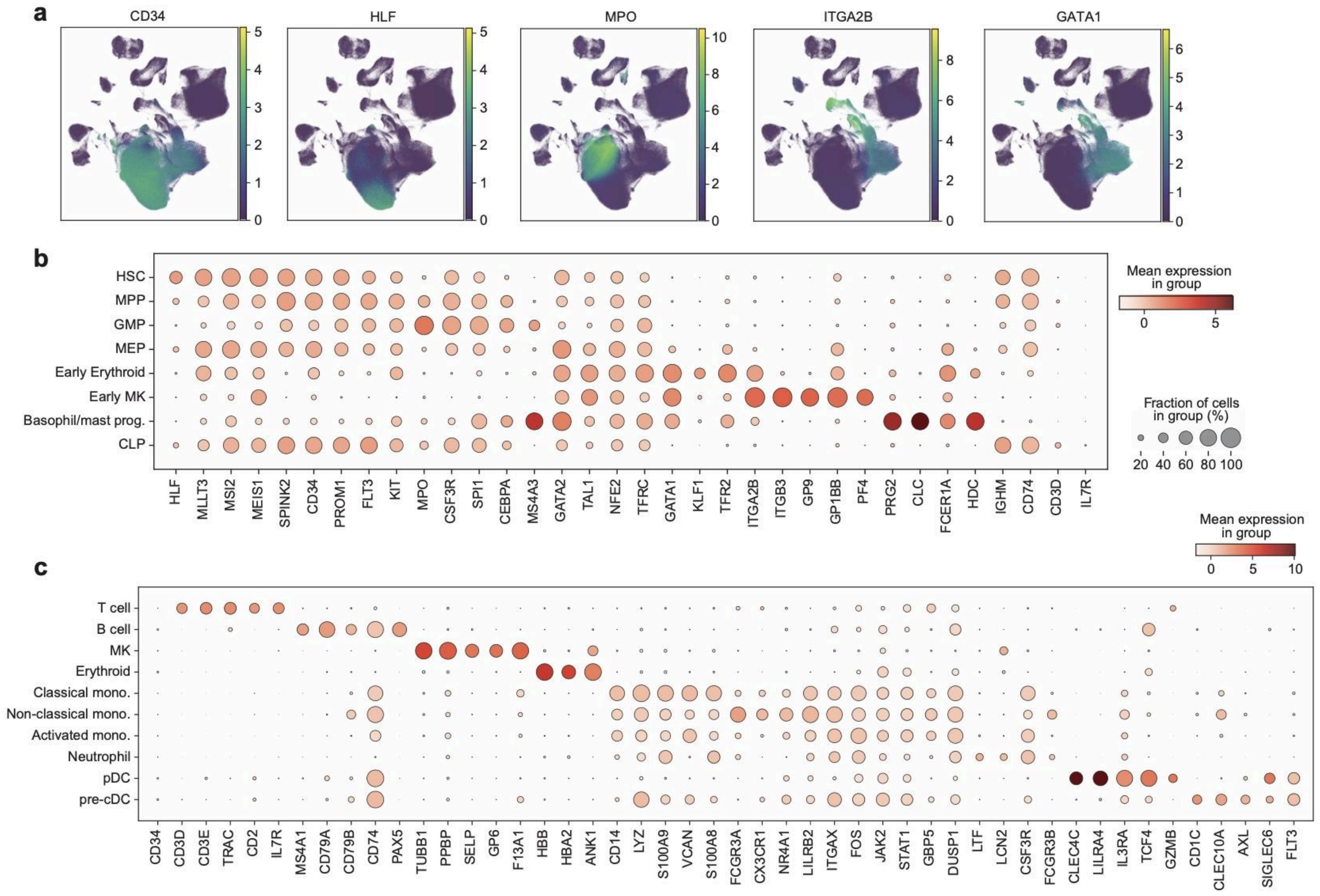

Supplementary Figure 7: Markers for cell typing in MPNs. a, UMAPs of patient-integrated scVI[59] latent space colored by log-normalized expression of selected hematopoiesis markers[28]. **b**, Z-scored log-normalized expression of selected markers of HSPC cell types. HSC, hematopoietic stem cell; MPP, multipotent progenitor; GMP, granulocyte-monocyte progenitor; MEP, megakaryocyte–erythroid progenitor; MK, megakaryocyte; CLP, common lymphoid progenitor. **c**, Z-scored log-normalized expression of selected markers of mature cell types. MK, megakaryocyte; pDC, plasmacytoid dendritic cell; cDC, conventional dendritic cell.

bioRxiv preprint doi: https://doi.org/10.64898/2026.04.11.717967; this version posted July 3, 2026. The copyright holder for this preprint (which was not certified by peer review) is the author/funder, who has granted bioRxiv a license to display the preprint in perpetuity. It is made available under a CC-BY 4.0 International license.

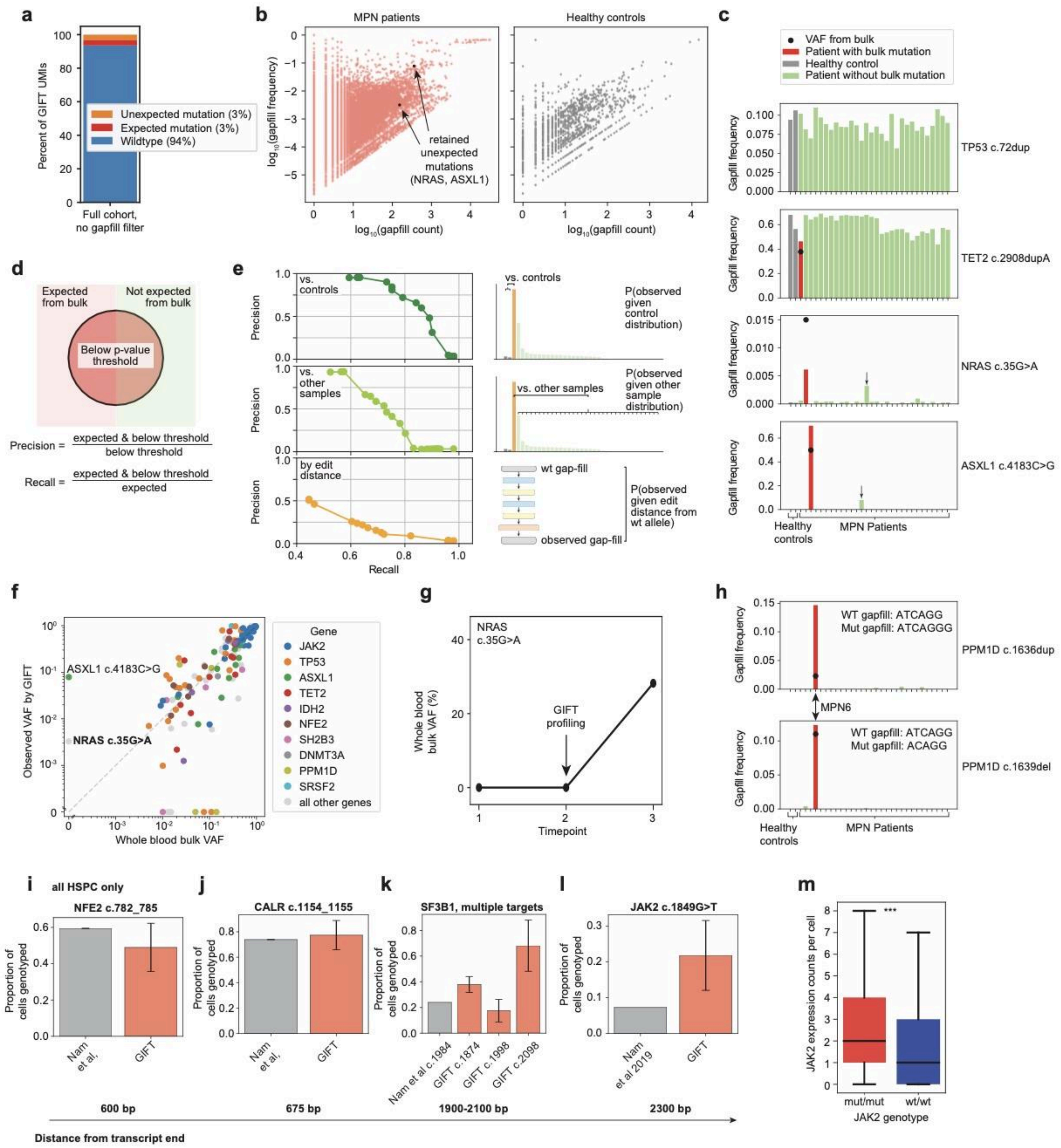

Supplementary Figure 8: Variant discovery and genotyping flexibility with GIFT in MPN cohort. a, Overview of observed gapfill sequences in the MPN cohort. Expected mutations are UMIs with gapfills exactly matching expected mutated alleles. Unexpected mutations are UMIs with gapfills that match neither wildtype nor expected mutation alleles. **b**, Frequency and count of gapfills with unexpected mutations in the MPN cohort. Gapfill count is the number of UMIs of that gapfill per patient, and gapfill frequency is the count normalized by the total observed UMIs for that targeted site (any gapfill sequence) for that patient. *Left*: all unexpected gapfills (n=38,344 across all patients). *Right*: unexpected gapfills observed in healthy controls

bioRxiv preprint doi: https://doi.org/10.64898/2026.04.11.717967; this version posted July 3, 2026. The copyright holder for this preprint (which was not certified by peer review) is the author/funder, who has granted bioRxiv a license to display the preprint in perpetuity. It is made available under a CC-BY 4.0 International license.

(n=2,988) to show scale of background noise. Sequencing saturation of GIFT for all patients is >95%. **c,** Representative observed gapfills and their frequencies across samples. The top two variants (*TP53*, *TET2*) have a high background in healthy controls. The bottom two variants (*NRAS*, *ASXL1*) were expected in two patients but also were significantly enriched in two other patients (arrows). **d-e**, Defining an empirical feature set using observed gapfills in the MPN cohort. **d**, Precision and recall definitions for testing how well each model distinguishes true variants (expected mutations) from noise (vast majority of unexpected gapfills). **e**, Precision-recall curves for three different models, which are detailed in methods, that can be used to assign the probability of observing each gapfill. As shown by the schematics on the right, the control model compares the observed gapfill frequency to that in healthy controls, the other sample model compares the observed gapfill frequency to that in other samples, and the edit distance model compares the observed gapfill frequency to the expected frequency if the true allele is wildtype as calculated in **Supplementary Fig. 6h-j**. **f**, VAF observed by GIFT vs. bulk whole blood, as in **Fig. 4f** but on a log scale. Labeled variants were not expected from bulk sequencing but were discovered as significant under the models shown in **e** using thresholds described in methods. In contrast, four variants were expected at VAF >= 0.05 but were not observed (2 *TP53*, 2 *PPM1D*). Three of these targets were missed because of low detection (probe counts <15), while one (*TP53* c.733G>A) was confirmed to have a lower VAF (0.01) in CD34+ cells than in whole blood (**Extended Data Table 2**). **g**, Longitudinal whole blood bulk VAF of *NRAS* c.35G>A showing that this mutation was not detected at the time of GIFT profiling but becomes prevalent in this patient at a subsequent timepoint. **h**, Frequencies of *PPM1D* mutations that can be captured by the same probe pair and gap. **i,** Proportion of cells genotyped by GIFT vs GoT (Nam et al)[10]**.** Variants or regions (*SF3B1*) are shown that were profiled in both studies. For variants near transcript ends (*NFE2*, *CALR*), GIFT capture is similar to GoT, while GIFT capture is better for variants far from transcript ends (*SF3B1*, *JAK2*) (GoT n=1; GIFT n=3, 14, 3, 21, 21, 36 profiled patients). Only HSPCs are included in calculations. **m**, *JAK2* expression in mutated vs wildtype cells. *JAK2* expression in *JAK2* V617F$^{mut/mut}$ cells is significantly higher than *JAK2* V617F$^{wt/wt}$ cells (n=138,090 mut cells, 91,513 wt cells, $p < 0.0005$, one-sided Mann-Whitney U).

bioRxiv preprint doi: https://doi.org/10.64898/2026.04.11.717967; this version posted July 3, 2026. The copyright holder for this preprint (which was not certified by peer review) is the author/funder, who has granted bioRxiv a license to display the preprint in perpetuity. It is made available under a CC-BY 4.0 International license.

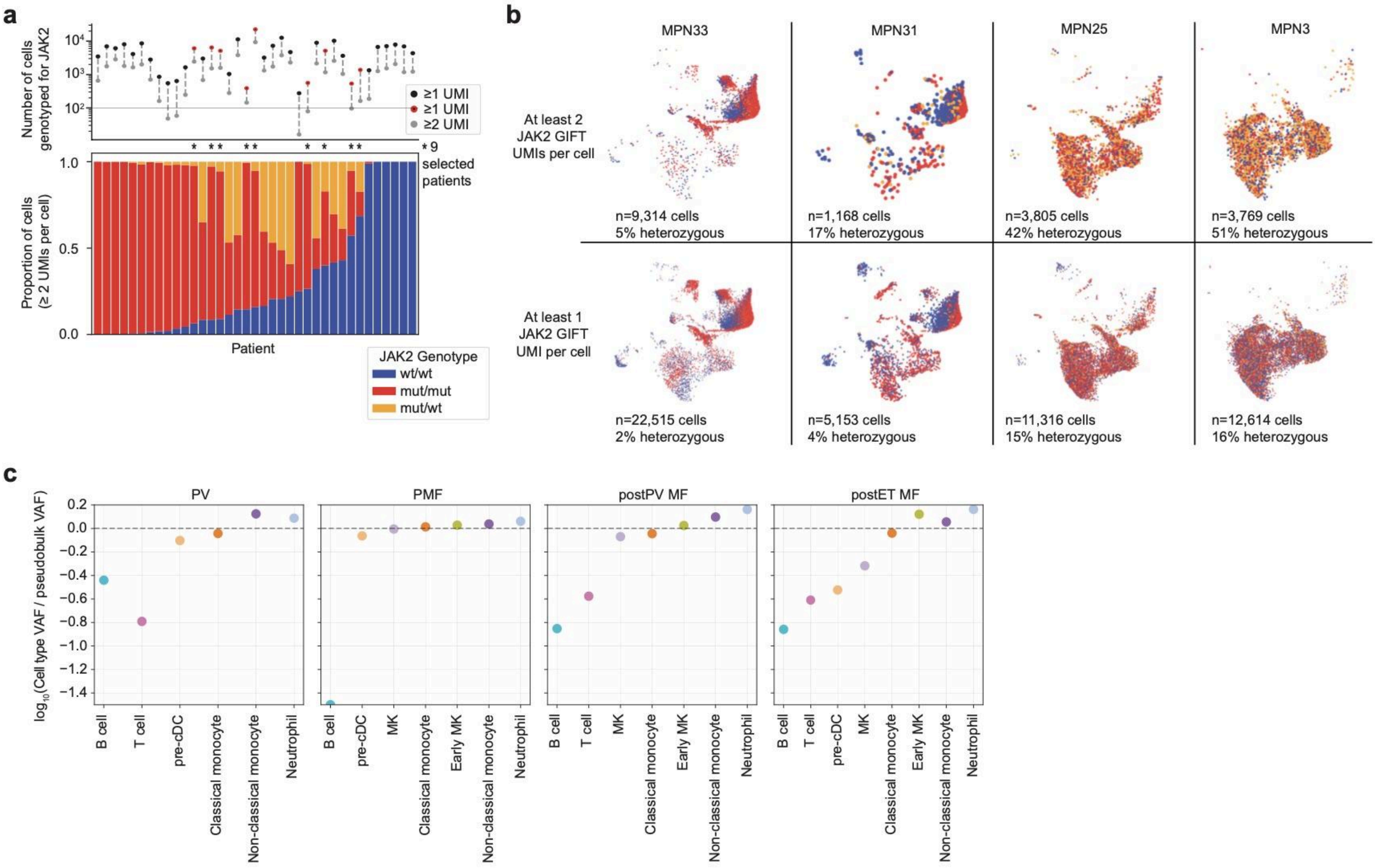

Supplementary Figure 9: JAK2 genotyping in MPNs. a, **a**, *Top*: Number of cells genotyped for *JAK2* V617F when including all cells with ≥ 1 UMI (*black* or *red*) or ≥ 2 UMIs (*grey*) for *JAK2* genotype. Red dots indicate patients selected as primarily homozygous (asterisks on bottom) and included in the MrVI integration used in **Fig. 5a-g**. *Bottom*: Proportion of cells from each patient that are called as homozygous wt (*wt*/*wt*), homozygous mutated (*mut*/*mut*), or heterozygous (*mut*/*wt*) for *JAK2* V617F including only cells with ≥ 2 *JAK2* UMIs. As shown on top, the number of genotyped cells varies widely by patient and is substantially reduced when requiring ≥ 2 UMIs versus ≥ 1 UMI (n cells per patient with ≥ 2 UMIs = 16 - 9,314 cells; n cells per patient with ≥ 1 UMI = 275 - 22,515 cells). **b**, JAK2 genotyping in 4 representative patients. Patients 1 and 2 are primarily wildtype or homozygous mutated so they are included in the patient set in **Fig. 5a-g**. Cells from each patient are shown on the UMAP from the patient-integrated scVI[59] latent space. **c**, Differential abundance of JAK2 V617F genotype by diagnosis. As in **Fig. 5c**, the median enrichment of the *JAK2* V617F mutation is shown for each cell type (normalized by pseudobulk VAF across all cell types), but here the values are split by patient diagnosis (n=1-3 patients per datapoint).

bioRxiv preprint doi: https://doi.org/10.64898/2026.04.11.717967; this version posted July 3, 2026. The copyright holder for this preprint (which was not certified by peer review) is the author/funder, who has granted bioRxiv a license to display the preprint in perpetuity. It is made available under a CC-BY 4.0 International license.

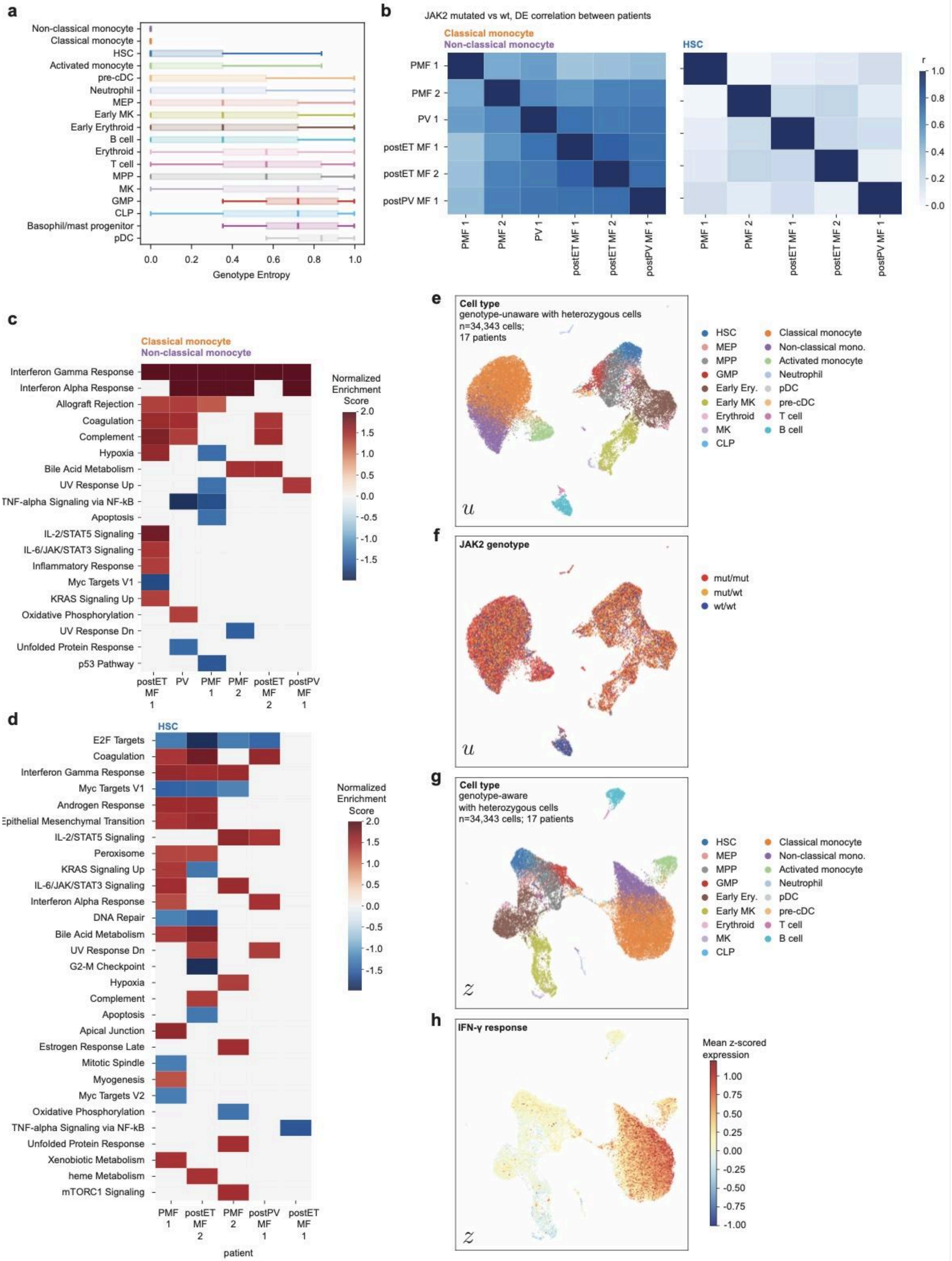

bioRxiv preprint doi: https://doi.org/10.64898/2026.04.11.717967; this version posted July 3, 2026. The copyright holder for this preprint (which was not certified by peer review) is the author/funder, who has granted bioRxiv a license to display the preprint in perpetuity. It is made available under a CC-BY 4.0 International license.

Supplementary Figure 10: *JAK2*-correlated differential expression across patients. a, Genotype entropy in MrVI genotype-aware z latent space, stratified by cell type. **b**, Pearson correlation matrices of differential gene expression (log fold-changes, $JAK2^{mut/mut}$ vs $JAK2^{wt/wt}$) across patients included in **Fig. 5g**. Each row/column is a patient and is labeled by diagnosis. **c-d**, Differentially expressed pathways by patient for the patients included in **Fig. 5g**. **e-h**, Additional UMAPs of MrVI latent spaces for the patient set that includes heterozygous *JAK2* mutation (cells in **Fig. 5h**). **e-f**, Genotype-unaware MrVI latent space (u) colored by cell type (**e**) or *JAK2* V617F genotype (**f**). **g-h**, Genotype-aware MrVI latent space (z) colored by cell type (**g**) or IFN-γ response gene score (**h**).

bioRxiv preprint doi: https://doi.org/10.64898/2026.04.11.717967; this version posted July 3, 2026. The copyright holder for this preprint (which was not certified by peer review) is the author/funder, who has granted bioRxiv a license to display the preprint in perpetuity. It is made available under a CC-BY 4.0 International license.

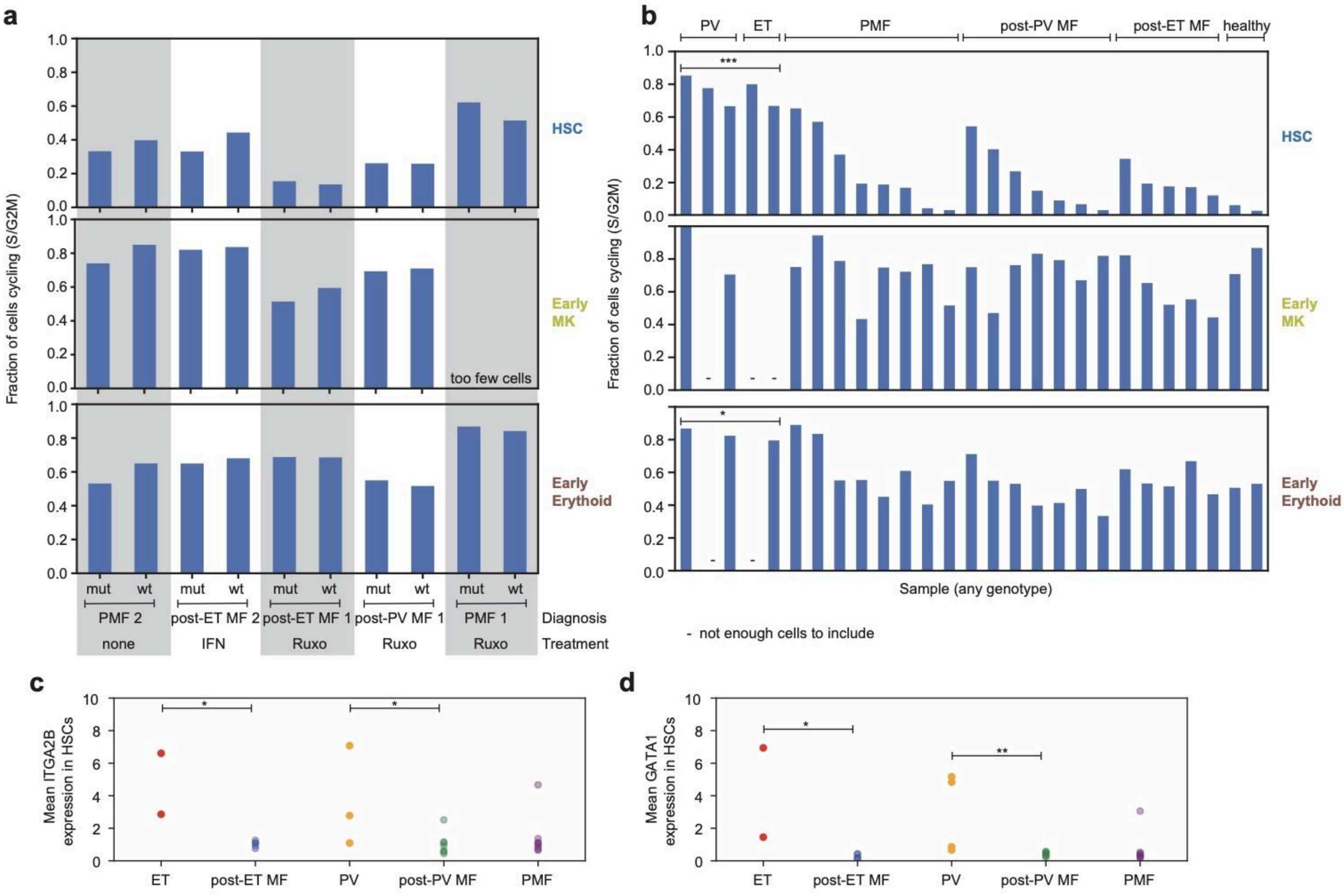

Supplementary Figure 11: HSPC proliferation and differentiation across MPN types. a, Quantification of cell cycling[76] in patients included in **Fig. 5g-ii**. Little difference is seen in the fraction of cells cycling (categorized as S or G2M phase) for $JAK2^{mut/mut}$ vs $JAK2^{wt/wt}$ cells. **b**, Fraction of cells cycling across all PV (polycythemia vera), ET (essential thrombocythemia), primary myelofibrosis (PMF), post-PV MF (post-polycythemia vera myelofibrosis) and post-ET MF (post-essential thrombocythemia myelofibrosis) patients. Healthy controls are shown for comparison. Patients with PV or ET have higher levels of cycling HSCs and early erythroid cells than MF patients (n=3-5 PV or ET, n=20 MF; one-sided Mann-Whitney U test; * $p<0.05$, *** $p<0.0005$). *JAK2* genotype is not considered for this broader patient set because patients may not have enough wildtype or mutated cells, and/or they have too many heterozygous cells, which confounds the analysis. **c-d**, Expression of megakaryocyte (*ITGA2B*) and erythroid (*GATA1*) markers in HSCs suggest greater differentiation bias in ET and PV HSCs than in secondary MF (n=3-8 patients per diagnosis, one-sided Mann-Whitney U test; * $p < 0.05$, ** $p < 0.005$).

bioRxiv preprint doi: https://doi.org/10.64898/2026.04.11.717967; this version posted July 3, 2026. The copyright holder for this preprint (which was not certified by peer review) is the author/funder, who has granted bioRxiv a license to display the preprint in perpetuity. It is made available under a CC-BY 4.0 International license.

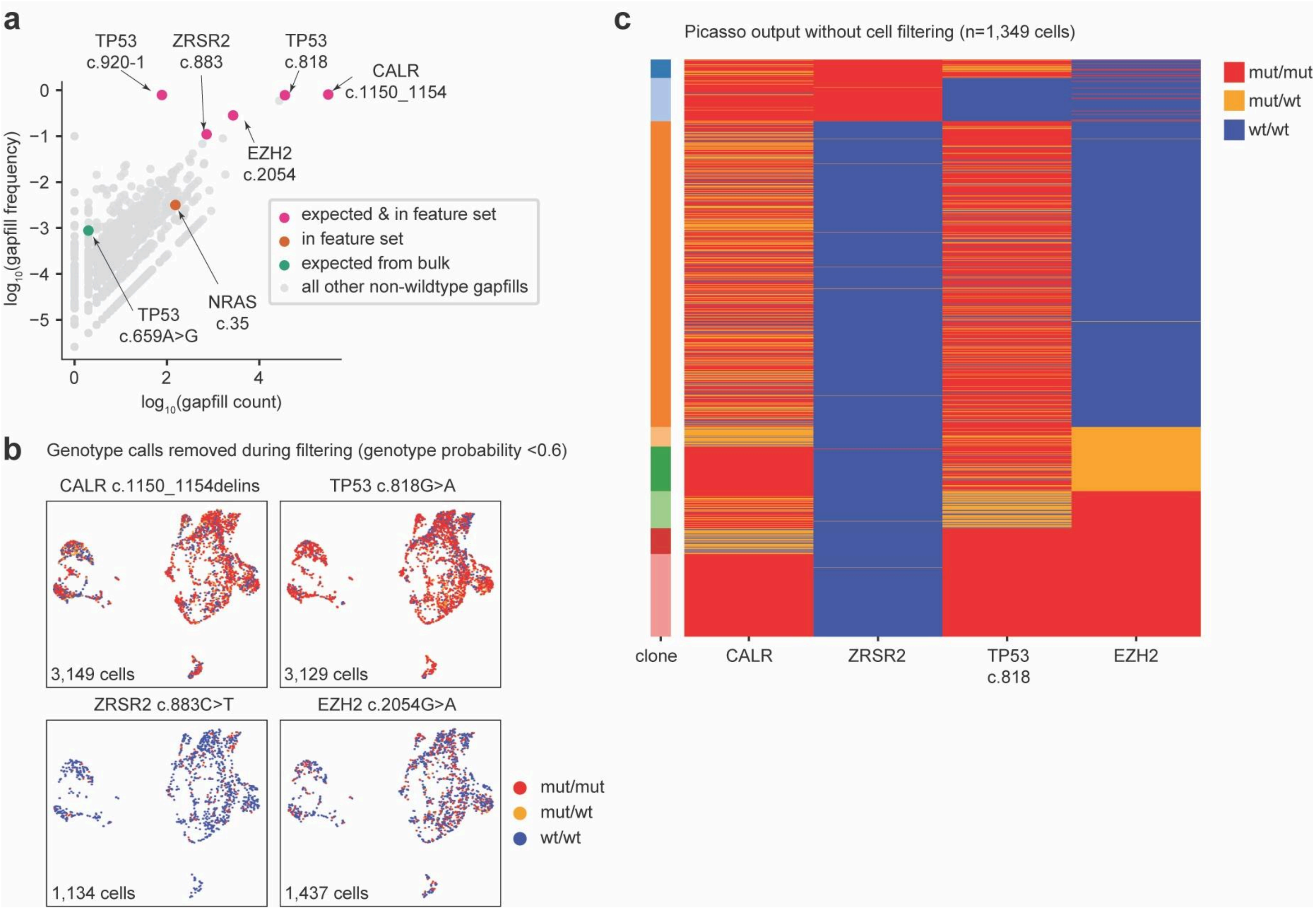

Supplementary Figure 12: Error model thresholds are necessary for a robust phylogeny. a, Frequency and count of gapfills observed in the transforming MPN patient. Frequency is the count normalized by the total observed UMIs for that targeted site (any gapfill sequence). Variants in pink were expected and significantly enriched in GIFT genotyping of this patient. We primarily consider the four of these with the most counts for phylogeny reconstruction. The *NRAS* mutation was discovered by our empirical feature set definition and validated by subsequent bulk sequencing. The *TP53* c.659A>G mutation was expected from whole blood bulk sequencing but not observed by GIFT profiling of the CD34^{+} compartment. Bulk sequencing confirmed a low frequency of this mutation in CD34^{+} cells (**Extended Data Table 2**). The grey dots show observed gapfills that are unlikely to be real mutations and were removed by our feature set definition. **b**, Genotype calls that were removed during filtering [P(genotype) < 0.6). The genotypes in **Fig. 6a** show the retained set [P(genotype) > 0.6]. Though 0.6 seems like a lenient threshold, the model considers homozygous mutation, heterozygous mutation, and wildtype as possible genotypes, so if P(mut/mut) is ~0.6, then typically the uncertainty reflects that the cell could be heterozygous [P(mut/wt) >0.3], while the probability that the cell is wildtype is low. **c**, Heatmap of genotypes by clone without probabilistic filtering. This heatmap is generated by PICASSO[44] as in **Supplementary Fig. 14a**. However, here we include cells genotyped at any confidence level, and we distinguish between heterozygous and homozygous mutation for all variants. Cells with genotypes for all 4 variants are included (n = 1,349 cells). With this unfiltered set of cells, PICASSO finds eight clones, and these are uninterpretable as a meaningful phylogeny.

bioRxiv preprint doi: https://doi.org/10.64898/2026.04.11.717967; this version posted July 3, 2026. The copyright holder for this preprint (which was not certified by peer review) is the author/funder, who has granted bioRxiv a license to display the preprint in perpetuity. It is made available under a CC-BY 4.0 International license.

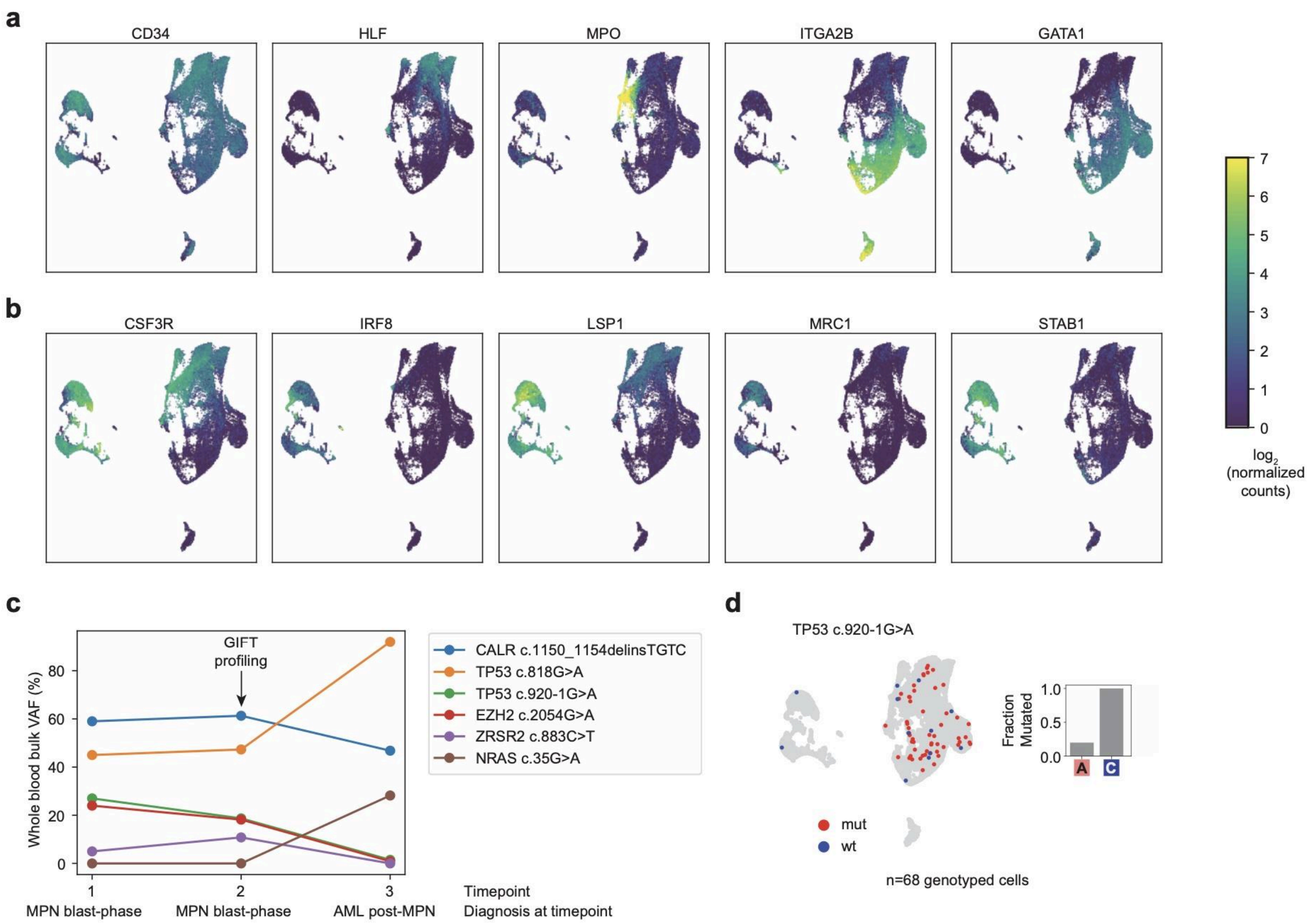

Supplementary Figure 13: Transformation to AML in an MPN patient. a, UMAP embedding of transforming MPN patient as in **Fig. 6** but colored by expression of HSPC marker genes[28]. The population labeled as leukemic blast in **Fig. 6b** expresses *CD34* but not these other canonical HSPC marker genes. **b**, The leukemic blast cells express myeloid markers, matching subsequent diagnosis as acute myeloid leukemia[28,74]. **c**, Whole blood bulk VAFs for this patient at 3 timepoints. The 2nd timepoint was profiled by GIFT. By the 3rd timepoint, the patient's disease had transformed to post-MPN AML. **d**, Genotyping of splice variant *TP53* c.920-1G>A. The splice variant is captured from pre-mRNA, so detection is low (n=68 genotyped cells), but the mutation is enriched in clone C. This is consistent with disappearance of this mutation at timepoint 3 (as shown in **c**).

bioRxiv preprint doi: https://doi.org/10.64898/2026.04.11.717967; this version posted July 3, 2026. The copyright holder for this preprint (which was not certified by peer review) is the author/funder, who has granted bioRxiv a license to display the preprint in perpetuity. It is made available under a CC-BY 4.0 International license.

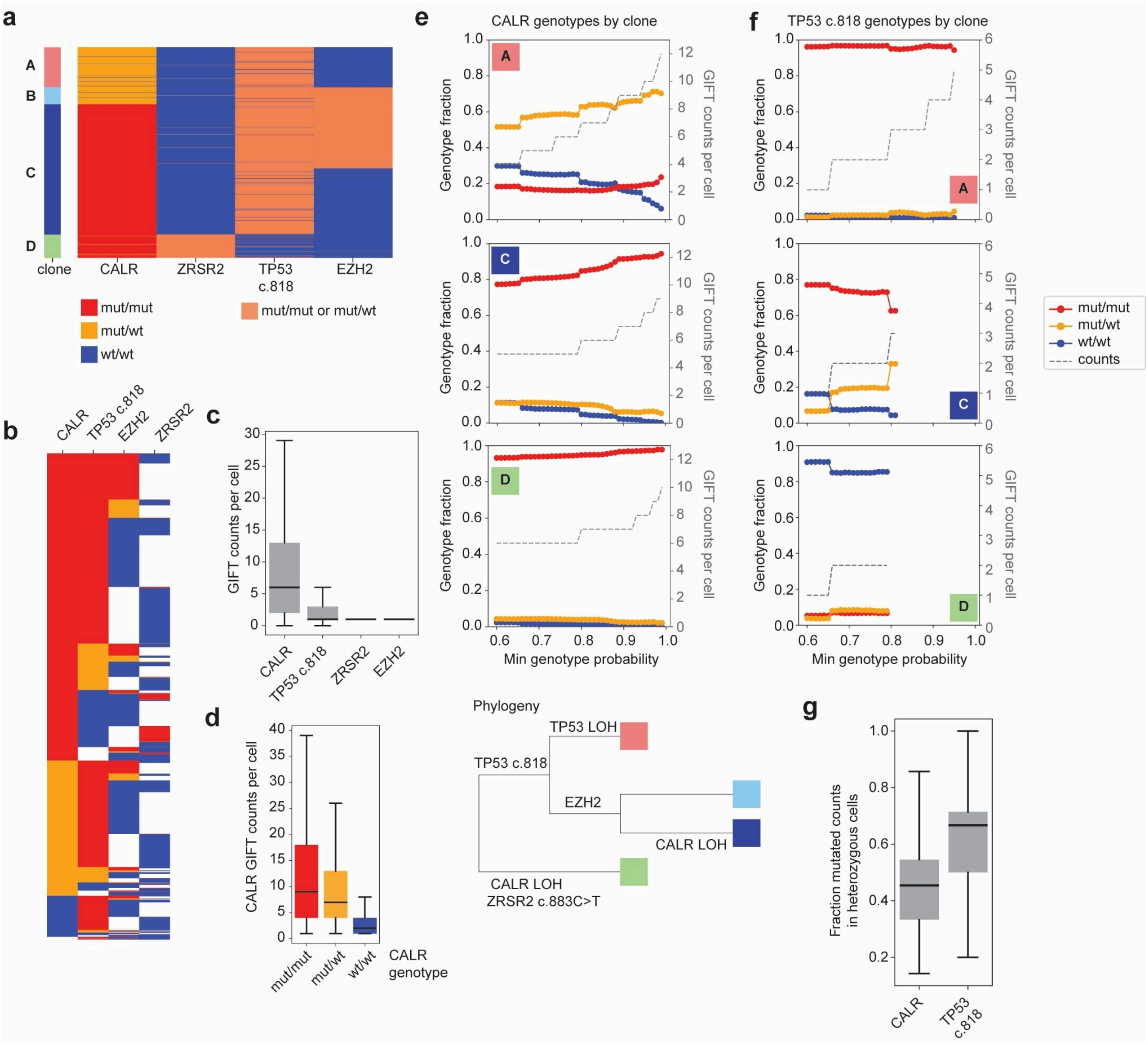

Supplementary Figure 14: Additional GIFT genotyping details for MPN patient transforming to AML. **a**, Heatmap of genotypes by clone as generated by PICASSO[44]. We had sufficient resolution to distinguish between *CALR* homozygous mutation (mut/mut) and heterozygous mutation (mut/wt) cells. For other variants, we group mut/mut and mut/wt cells together for phylogeny inference by PICASSO. Only cells genotyped for all 4 variants are included (n=730 cells). We manually merged two clones to form clone C because they were artificially separated by *EZH2* wildtype versus mutated calls for cells that are all likely heterozygous. **b**, Heatmap of genotypes sorted by genotype rather than clone and including all cells genotyped for at least 3 variants (n=5,398 cells). Here we distinguish mut/mut and mut/wt calls for all variants to more fully show the underlying data. **c**, GIFT counts per cell by targeted variant (n = 27,180; 16,845; 5,230; 6,894 genotyped cells per variant from left to right). **d**, *CALR* GIFT counts per cell by *CALR* genotype. *CALR* mutation is correlated with higher *CALR* GIFT counts per cell ($CALR^{mut/mut} > CALR^{mut/wt} > CALR^{wt/wt}$; $p < 0.0005$, one-sided Mann-Whitney U test; n = 3,348-14,726 cells). **e**, *CALR* genotyping by clone at different genotype probability thresholds. Clones B and C are predominantly $CALR^{mut/mut}$, especially

bioRxiv preprint doi: https://doi.org/10.64898/2026.04.11.717967; this version posted July 3, 2026. The copyright holder for this preprint (which was not certified by peer review) is the author/funder, who has granted bioRxiv a license to display the preprint in perpetuity. It is made available under a CC-BY 4.0 International license.

in high confidence cells. **f**, *TP53* genotyping by clone at different genotype probability thresholds. Cells in clone A are *TP53*$^{mut/mut}$. More cells in clone B are *TP53*$^{mut/mut}$ than *TP53*$^{mut/het}$, but this is biased by higher expression of the mutated *TP53* allele than wildtype allele, as shown in **g**. **g**, Fraction of counts that are mutated for cells that are categorized as heterozygous for *CALR* or *TP53* (most likely genotype by GIFT probabilistic model). Mutated counts are enriched over wildtype counts in *TP53* heterozygous cells ($p=1.8*10^{-81}$; one-sided binomial test, n = 5,337 *TP53*-heterozygous cells; n=62,484 *CALR*-heterozygous cells).

bioRxiv preprint doi: https://doi.org/10.64898/2026.04.11.717967; this version posted July 3, 2026. The copyright holder for this preprint (which was not certified by peer review) is the author/funder, who has granted bioRxiv a license to display the preprint in perpetuity. It is made available under a CC-BY 4.0 International license.

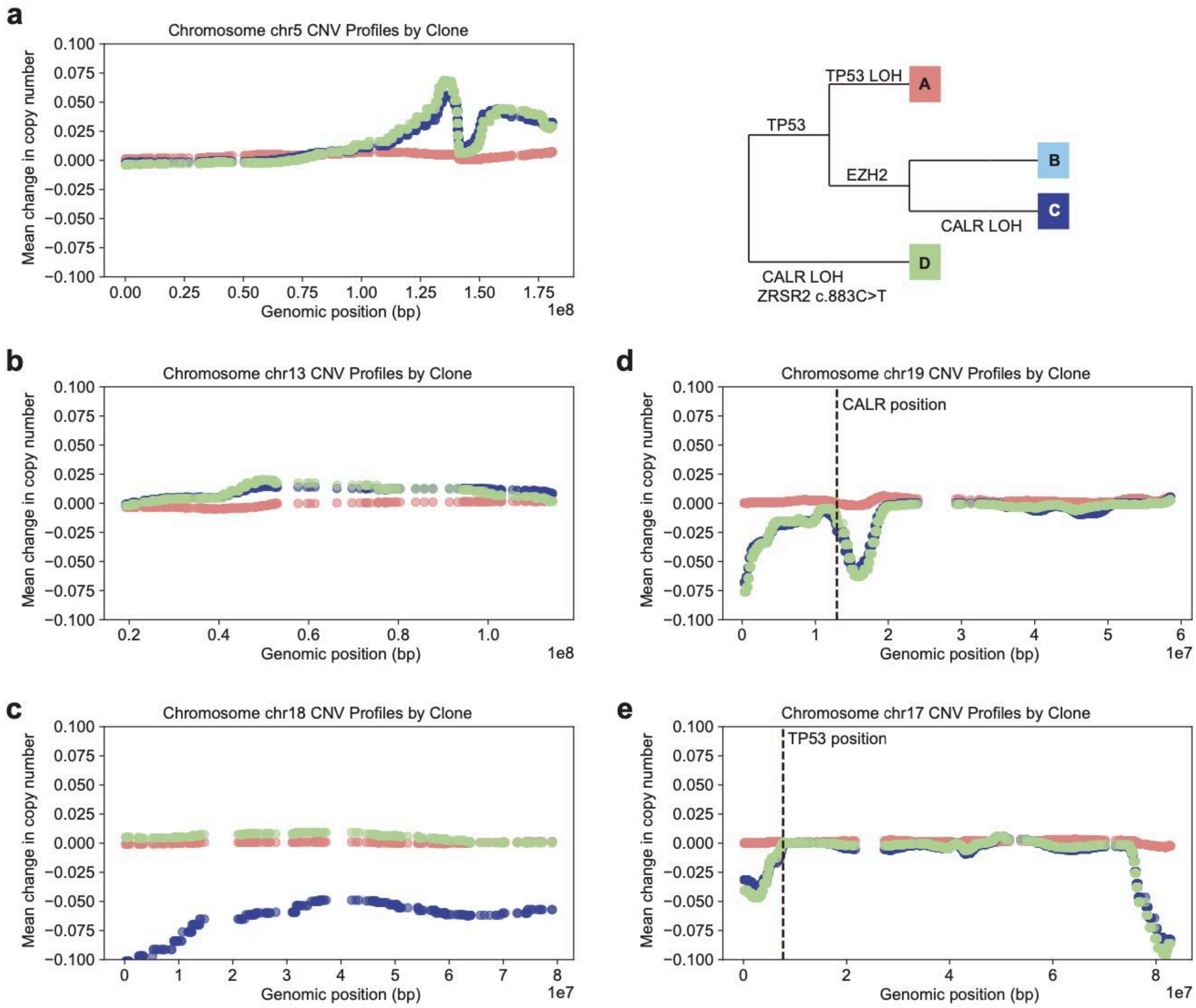

Supplementary Figure 15: Inferred copy number variants for MPN patient transforming to AML. a-c, Inferred CNV profiles from single-cell gene expression. Clone A was used as the copy number reference in all panels. Clone A shows loss of chromosome 5q and chromosome 13q (**a**, **b**), while clone C shows loss of chromosome 18 (**c**). These CNVs were also found independently by clinical karyotyping (**Extended Data Table 2**). **d**, Inferred CNV profile for chromosome 19 showing copy number loss around *CALR* for clones C and D. This is consistent with *CALR* LOH in these clones, as inferred by GIFT genotyping (**Supplementary Fig. 14e**). No CNV was found by clinical karyotyping of chromosome 19 (**Extended Data Table 2**). **e**, Inferred CNV profile for chromosome 17, which contains *TP53*. There is no indication of chromosome loss near TP53 for clone A, despite strong evidence of LOH by genotyping (**Supplementary Fig. 13c**; **Supplementary Fig. 14f, Extended Data Table 2**).

bioRxiv preprint doi: https://doi.org/10.64898/2026.04.11.717967; this version posted July 3, 2026. The copyright holder for this preprint (which was not certified by peer review) is the author/funder, who has granted bioRxiv a license to display the preprint in perpetuity. It is made available under a CC-BY 4.0 International license.

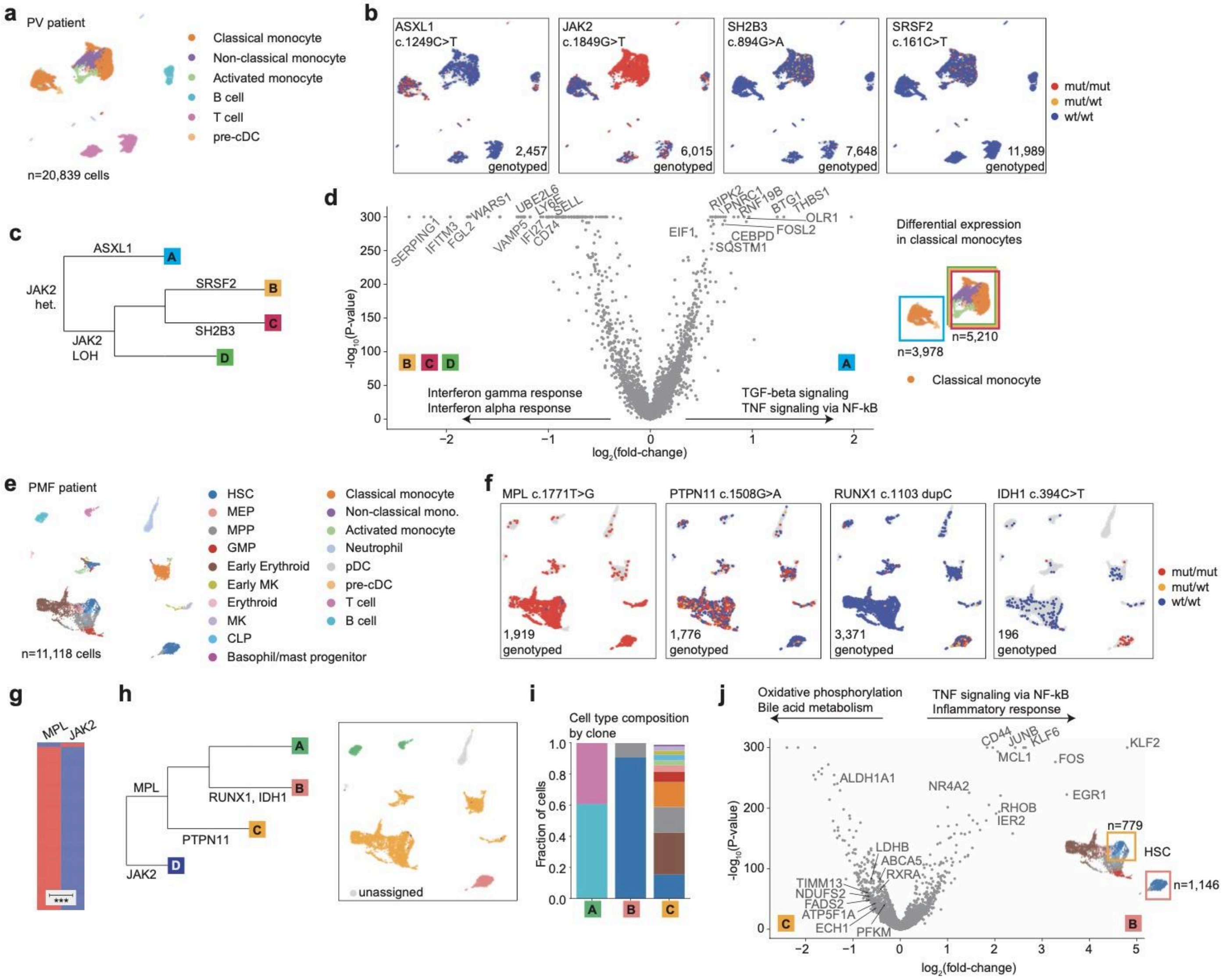

Supplementary Figure 16: Lineage tracing of additional MPN patients. a-d, Genotyping and lineage tracing of an individual patient in the MPN cohort with polycythemia vera (PV). **a-b**, UMAP embedding of this individual patient with cells colored by cell type (**a**) or GIFT genotype for 4 different variants (**b**). **c**, Inferred phylogeny using PICASSO[44]. LOH is inferred by mutation frequencies in each clone. **d**, *Left:* Volcano plot showing differential expression[61] in classical monocytes of clone A vs. clones B-D (n = 12,066 genes). Significantly enriched pathways are shown for each clone with top pathway genes labeled (FDR < 0.1, methods)[62]. *Right*: Region of UMAP used for differential expression analysis is shown. Only classical monocytes were included in differential expression analysis. **e-j**, Genotyping and lineage tracing of an individual patient in the MPN cohort with primary myelofibrosis (PMF). **e-f**, UMAP embedding of this patient with cells colored by cell type (**e**) or GIFT genotype for 4 different variants (**f**). **g**, Heatmap showing mutually exclusive mutation of *MPL* and *JAK2*, which allows us to separate these mutations into separate clones (Fisher's exact test, p = $1.3*10^{-13}$; n = 317 cells genotyped for both variants). **h**, *Left:* Inferred phylogeny using PICASSO[44] and manual annotation. We included *MPL*, *RUNX1*, and *PTPN11* mutations for PICASSO (n = 275 cells genotyped for all 3 variants), and then we manually added *JAK2* and *IDH1* mutations based on mutation co-occurrence (Fisher's exact test, as in **g**). *Right:* UMAP embedding colored by clone. Grey indicates wildtype or undetermined clone. **i**, Cell type composition by clone. Colors correspond to the legend in **e**. **j**, *Left:* Volcano plots showing differential expression[61] in HSCs of clone B versus clone C (n = 12,604 genes). The most significantly enriched pathways are shown for each clone with top pathway genes labeled

bioRxiv preprint doi: https://doi.org/10.64898/2026.04.11.717967; this version posted July 3, 2026. The copyright holder for this preprint (which was not certified by peer review) is the author/funder, who has granted bioRxiv a license to display the preprint in perpetuity. It is made available under a CC-BY 4.0 International license.

(FDR < 0.1, methods)[62]. *Bottom Right*: Cells used for differential expression analysis are indicated by boxes on UMAP.
